# Clinical study of renal damage in patients with Type-1 diabetic nephropathy based on shear wave elastography and hemodynamics

**DOI:** 10.12669/pjms.41.7.10863

**Published:** 2025-07

**Authors:** Wei Dang, Xingxian Li, Huidi Wei, Hui-ling Feng, Kang-yan Yu

**Affiliations:** 1 Wei Dang Department of Ultrasound, The People’s Hospital of Qingyuan District, Baoing 071000, Hebei, China; 2 Xingxian Li Department of Ultrasound, The People’s Hospital of Qingyuan District, Baoing 071000, Hebei, China; 3 Huidi Wei Department of Ultrasound, The People’s Hospital of Qingyuan District, Baoing 071000, Hebei, China; 4 Hui-ling Feng Department of Ultrasound, The People’s Hospital of Qingyuan District, Baoing 071000, Hebei, China; 5 Kang-yan Yu Department of Ultrasound, The People’s Hospital of Qingyuan District, Baoing 071000, Hebei, China

**Keywords:** Hemodynamic Testing, Type-1 Diabetic Nephropathy, Shear Wave Elastography

## Abstract

**Objective:**

To evaluate the clinical value of shear wave elastography(SWE) and hemodynamics in renal damage in patients with Type-1 diabetic nephropathy.

**Method:**

This was a retrospective study. One hundred and ten patients with Type-1 diabetic nephropathy in The People’s Hospital of Qingyuan District from January 2021 to January 2024 were selected as the study group, while sixty healthy subjects in the same period were selected as the healthy control group. The elasticity indexes of renal cortex and medulla in two groups were compared, as well as the hemodynamic indexes of different severity of patients.

**Result:**

The parameters such as Young’s modulus E value and shear wave velocity in the renal cortex and medulla in the study group were significantly higher than those in the control group (p=0.00). Young’s modulus (E values) in the renal cortex and medulla in patients with diabetic nephropathy was positively correlated with urinary protein, serum creatinine, and serum uric acid concentration (p=0.00). Peak systolic velocity(PS), peak diastolic velocity(ED), mean velocity(TAMx), and minimum velocity(TAMn) in the arteries of kidney of the control group and early-stage group were significantly higher than those in the mid and late-stage groups (p=0.00), while resistive index(RI) was significantly lower (p=0.00).

**Conclusion:**

SWE and hemodynamic testing may evaluate the severity of renal damage in patients with Type-1 diabetic nephropathy in a non-invasive manner, thereby providing references for dynamic monitoring of disease progression, evaluating the effect of clinical intervention, and adjusting treatment regimens.

## INTRODUCTION

In recent years, the incidence of Type-1 diabetes has increased substantially, seriously threatening the health of patients. Diabetic renal impairment is a major microvascular complication of diabetes that gradually progresses to end-stage renal disease.^1^ Currently, the common approaches for the clinical judgment of renal damage include measurement of biological indicators such as serum creatinine, urea nitrogen, and urine protein, as well as renal color Doppler ultrasonography, which are primarily used for the diagnosis after the occurrence of renal damage but fail to predict the risk of renal damage in patients with Type-1 diabetes.^2^

Notably, the kidney has a strong compensatory function, and despite no significant changes in commonly used clinical biochemical indicators when the damaged nephron accounts for less than 75% of all nephrons, there may be injurious changes in the glomeruli at this time. While pathological changes are considered the gold standard for the diagnosis of diabetic nephropathy(DN), they involve invasive procedures, making it difficult for most patients to undergo renal biopsy, and it is not suitable to dynamically monitor the development of the disease due to erroneous sampling, thus limiting its clinical application to some extent. By contrast, SWE, as a noninvasive detection technique, can be used to assess the tissue elasticity and stiffness by measuring Young’s modulus of the tissue.^3^

Moreover, the renal artery RI is a sensitive indicator for predicting the complications of microvascular nephropathy in Type-1 diabetes.^4^ In this study, the renal damage in patients with Type-1 diabetic nephropathy was evaluated using SWE and hemodynamics, so as to provide references for the early diagnosis of DN, and evaluate the clinical value of shear wave elastography(SWE) and hemodynamics in renal damage in patients with Type-1 diabetic nephropathy.

## METHODS

This was a retrospective study. One hundred and ten patients with Type-1 diabetic nephropathy in The People’s Hospital of Qingyuan District from January 2021 to January 2024 were selected as the study group and divided into the early, mid, and late stages according to puncture pathology and clinical severity, while sixty healthy subjects in the same period were selected as the healthy control group.

### Ethical approval:

The study was approved by the Institutional Ethics Committee of the People’s Hospital of Qingyuan District (No.:2020-121; date: January 21, 2020), and written informed consent was obtained from all participants or the guardian of the participants.

### Inclusion criteria:

- Patients who met the diagnostic criteria of Type-1 diabetes mellitus and were confirmed as combined with kidney disease by renal biopsy.^5^,^6^
- Patients who gave informed consent.
- Patients with complete clinical data.
- Patients with clear consciousness, no mental system diseases.
- Patients aged > 14 years, generally in good condition.

### Exclusion criteria:

- Patients with acute infectious diseases such as urinary tract infection or local skin lesions.
- Patients combined with other renal diseases.
- Patients with mental illness or other cognitive impairment who couldn’t cooperate to complete the study.
- Patients with primary glomerular disease or other secondary glomerular disease.
- Patients who had started receiving renal replacement therapy during renal biopsy.

Patients with Type-1 diabetes mellitus complicated with nephropathy in our hospital were selected as the study group and divided into the early stage(with urinary albumin excretion<200 μg/min), mid-stage(with urinary protein excretion>200 μg/min), and late stage(with decline of renal function progressing to end-stage renal disease and glomerular filtration rate dropping to below 15 ml/min) according to puncture pathology and clinical severity.^7^ In the meantime, sixty healthy subjects in the same period were selected as the healthy control group. No significant differences were observed in general data between the two groups (Table-I).

**Table-I:** Comparative analysis of general data between the study and control groups (*χ̅*±*S*).

Indicator	Study Group	Control Group	t/χ^2^	P
n	110	60		
Age (years)	25.53±8.76	26.68±7.53	0.86	0.39
M (n %)	57(52%)	32(53%)	0.02	0.88
BMI (kg/m^2^)	22.54±3.21	22.42±3.13	0.23	0.82

P>0.05

### Instruments and test methods:

#### 1) Shear wave elastography:

Color Doppler ultrasound diagnostic apparatus (Supersonic Imagine AixPlorer, France) was used. The subjects were placed in the right lateral decubitus position, with the size, shape, and internal structure of the left kidney observed using the conventional ultrasound. After stabilizing the images, the subjects were instructed to hold their breath, followed by activating the SWE imaging mode. The Q-box was placed in the renal parenchyma area, with the system automatically calculating the mean elastic modulus(Emean), maximum(Emax), and minimum(Emin) of the renal parenchyma in the Q-box area. Notably, the above indicators were measured repeatedly for three times to obtain an average value. In SWE images, red shows hard tissue in the lesion while blue means soft tissue, and the higher value of elastic modulus indicates greater tissue stiffness.^8^ All procedures were performed by a senior sonographer.

#### 2) Renal hemodynamic testing:

The abdominal convex array probe was used, with the subjects placed in a supine or lateral decubitus position. The time-flow spectrum and hemodynamic parameters were obtained by sampling the main renal artery of the hilum, the segmental artery of the renal sinus, and the proximal interlobar artery using Pulsed Doppler. Each vessel was measured three times to obtain the average of the parameters measured in each of the three vessels. All procedures were performed by the same sonographer.

#### 3) Laboratory tests:

Ten ml of fasting venous blood was collected from all patients. Urinary microalbumin, serum creatinine, and uric acid were measured by a Hitachi 7600 automatic biochemical analyzer.

### Statistical analysis:

SPSS 20.0 software was used for the statistical analysis of all data, and measurement data were expressed as(*χ̅*±*S*). Inter-group data were analyzed using the t-test, the analysis of variance(AOV) was utilized for the multi-group data analysis, and rates were compared using the c^2^ test. Correlations were expressed using Pearson correlation coefficients, and P<0.05 was considered statistically significant.

## RESULTS

The parameters of Emean, Emax, Emin, and shear wave velocity in the renal cortex and medulla of patients were significantly higher in the study group than in the control group (p=0.00, Table-II). Young’s modulus and shear wave velocity of renal cortex and medulla in early and middle stage DN patients were significantly lower than those in advanced stage (P=0.00, Table-III). The elasticity indexes of different grades of glomerulosclerosis in diabetic nephropathy suggested that Young’s modulus and shear wave velocity in renal cortex and medulla of grades III and IV patients were considerably higher than those of grades I and II patients(P=0.00, Table-IV). There is a positive correlation between Young’s modulus Emean and Emax values and urinary protein, serum creatinine, and serum uric acid concentrations in the renal cortex and medulla of DN (P=0.00, Table-V). PS, ED, TAMx, and TAMn of the renal artery, segmental artery of the renal sinus, and interlobar artery in the control and early-stage groups were significantly higher than those in the mid and late-stage groups, while RI was substantially lower (p=0.00, Table-VI).

**Table-II:** Comparative analysis of elasticity indexes between the diabetic nephropathy and healthy control groups (*χ̅*±*S*).

Indicator		Study Group	Control Group	t	p
n		110	60		
Young’s modulus and shear wave velocity of renal cortex (m/s)	Emean*	17.83±4.72	11.20±3.87	9.30	0.00
	Emax*	32.07±11.36	21.84±9.08	6.63	0.00
	Emin*	11.93±3.75	8.11±2.38	7.26	0.00
	Shear wave velocity*	3.26±0.54	1.22±0.48	24.46	0.00
Young’s modulus and shear wave velocity of renal medulla (m/s)	Emean*	15.08±4.37	10.49±3.32	8.47	0.00
	Emax*	23.18±9.03	15.68±5.31	5.57	0.00
	Emin*	8.95±3.04	5.27±2.26	8.68	0.00
	Shear wave velocity*	3.12±0.63	1.02±0.17	24.08	0.00
Young’s modulus and shear wave velocity of renal sinus (m/s)	Emean	14.32±5.74	13.85±6.03	0.26	0.80
	Emax	22.68±7.19	22.89±6.80	0.18	0.85
	Emin	5.68±1.03	5.53±1.10	0.89	0.38
	Shear wave velocity*	2.07±0.64	2.13±0.52	0.62	0.53

* P<0.05

**Table-III:** Comparative analysis of elasticity indexes in different stages of DN (*χ̅*±*S*) n=110.

Stage	N	Renal cortex		Renal medulla	
		Young’s modulus (Kpa)*	Shear wave velocity (m/s)*	Young’s modulus (Kpa)*	Shear wave velocity (m/s)*
Early	45	12.37±1.26	1.72±0.33	11.06±1.15	1.36±0.40
Mid	37	14.83±2.07	2.46±0.61	12.79±1.24	1.80±0.62
Late	28	17.73±2.73	3.04±0.58	14.70±1.43	2.37±0.74
F		9.62	9.46	9.11	5.61
p		0.00	0.00	0.00	0.00

* P<0.05

**Table-IV:** Comparative analysis of elasticity indexes of different grades of glomerulosclerosis (*χ̅*±*S*) n=110.

Grade of glomerulosclerosis	n	Renal cortex		Renal medulla	
		Young’s modulus (Kpa)*	Shear wave velocity (m/s)*	Young’s modulus (Kpa)*	Shear wave velocity (m/s)*
Grade 1	46	11.04±1.20	1.53±0.36	10.32±2.02	1.34±0.42
Grade 2	33	12.28±1.14	1.80±0.47	11.08±1.98	1.76±0.38
Grade 3	19	14.63±1.63	2.32±0.40	12.37±2.33	2.07±0.52
Grade 4	12	15.53±2.01	2.68±0.51	14.09±2.15	2.45±0.38
*F*		9.32	8.56	5.38	13.06
*p*		0.00	0.00	0.00	0.00

* P<0.05

**Table-V:** Analysis of correlation between Young’s modulus (E values) and laboratory test indicators (r).

Laboratory indicators	Examined site	Young’s modulus (E values)	Correlation	
			r	p
Creatinine (umol/L)	Cortex	Emean*	0.436	0.00
		Emax*	0.679	0.00
	Medulla	Emean*	0.593	0.00
		Emax*	0.576	0.00
Uric acid (umol/L)	Cortex	Emean*	0.488	0.00
		Emax*	0.672	0.00
	Medulla	Emean*	0.570	0.00
		Emax*	0.592	0.00
Urine protein (μg/min)	Cortex	Emean*	0.489	0.00
		Emax*	0.620	0.00
	Medulla	Emean*	0.547	0.00
		Emax*	0.603	0.00

* P<0.05

**Table-VI:** Analysis of correlation between Young’s modulus (E values) and laboratory test indicators (r).

Parameter	Late-stage	Mid-stage	Early-stage	Control group	F	p
n	28	37	45	60		
***Renal artery***						
PS (cm/s)	67.35±7.06	69.73±6.78	73.44±10.37	79.08±13.78	12.06	0.00
ED (cm/s)	13.58±2.23	14.50±2.35	21.47±4.86	26.39±5.02	8.53	0.00
TAMx (cm/s)	38.29±6.63	42.73±7.15	50.28±7.40	53.17±8.02	7.66	0.00
TAMn (cm/s)	18.04±5.38	22.36±5.26	24.40±6.07	25.76±5.71	11.27	0.00
RI	0.74±0.04	0.72±0.08	0.68±0.04	0.65±0.03	7.83	0.00
***Segmental artery of the renal sinus***						
PS (cm/s)	43.26±5.76	45.09±5.31	50.40±5.90	54.11±6.42	9.60	0.00
ED (cm/s)	10.26±1.20	13.97±2.13	19.40±4.23	22.08±3.10	14.53	0.00
TAMx (cm/s)	25.92±4.07	28.56±5.27	32.54±5.70	36.93±6.22	17.04	0.00
TAMn (cm/s)	15.70±4.01	17.33±5.02	21.97±4.27	35.21±6.73	14.80	0.00
RI	0.78±0.06	0.72±0.08	0.64±0.07	0.60±0.04	17.34	0.00
***Interlobar artery***						
PS (cm/s)	26.83±4.54	30.24±4.62	35.76±3.67	38.17±4.81	12.37	0.00
ED (cm/s)	8.57±2.25	11.16±2.46	14.08±3.20	16.38±3.25	7.64	0.00
TAMx (cm/s)	18.43±4.21	22.89±4.18	24.07±5.21	26.40±5.32	11.49	0.00
TAMn (cm/s)	10.25±2.25	14.76±3.20	15.08±4.16	16.04±4.62	8.75	0.00
RI	0.88±0.10	0.75±0.12	0.69±0.06	0.60±0.08	9.15	0.00

P<0.05

## DISCUSSION

The findings of this study showed that the PS, ED, TAMx, and TAMn values of the renal arteries, segmental arteries of the renal sinus, and interlobar arteries in the control and early-stage groups were significantly higher than those in the mid and late-stage groups. It may be because high blood sugar aggravates glomerular and renal vascular sclerosis, and the basement membrane of the capillary network continues to thicken, leading to pathological damage of the kidney. This will cause hemodynamic changes at all levels of the renal artery, thereby reducing the flow rate of blood through the kidney, so that color Doppler ultrasound can effectively evaluate the change of renal function.^9^

This study also revealed a significantly lower RI in the control and early-stage groups than in the mid and late-stage groups, suggesting an elevated forward resistance in renal blood flow due to arterial sclerosis lesions. It has been reported that changes in the hemodynamic parameters of the renal arteries at various levels are more pronounced in diabetic patients, regardless of renal dysfunction, and color Doppler ultrasound can assess the renal blood perfusion status and hemodynamic changes and can even detect these changes in the early stages, which is consistent with the conclusion of this study.^10^ The measurement of the renal artery blood flow RI through renal ultrasound can effectively reflect its correlation with glomerulosclerosis and arteriolosclerosis, making it valuable for the early diagnosis of DN.^11^ Previous studies on SWE and hemodynamic detection in chronic kidney disease, especially diabetic nephropathy, are few, which proves the innovation of this study, and the conclusion of this study adds rare clinical data to this research field.

In recent years, the incidence of Type-1 diabetes has increased substantially among adolescents, posing a serious threat to their health and growth development.^12^ The disease is prone to causing damage to small blood vessels and nerves, resulting in a series of severe complications such as diabetic nephropathy, fundus disease, neurological complications, and diabetic gangrene.^13^ In patients with diabetic nephropathy, renal function gradually declines until end-stage renal disease, when the glomerular filtration rate drops below 15 ml/min and renal replacement therapy is often required.^14^

Therefore, early and accurate assessment of renal damage complications in patients with Type-1 diabetes, along with targeted preventive measures, is crucial for improving the prognosis of patients. Renal biopsy, despite being the gold standard for the diagnosis of DN, is an invasive procedure and is currently limited to cases where the cause of chronic kidney disease is difficult to identify. By contrast, SWE, a novel ultrasound elastography technique developed in recent years, overlays tissue elasticity imaging information on conventional ultrasound, allowing for the quantitative analysis and measurement of Young’s modulus of tissues and organs, and assesses the tissue stiffness using Young’s modulus. Research has shown that SWE is not influenced by the operator, with good repeatability and no need for external pressure, thus providing stable results with high diagnostic efficacy.^15^,^16^

There were few reports on clinical renal SWE imaging. This study confirmed that parameters such as Young’s modulus and transverse wave velocity of renal cortex and medullary tissue in DN patients were significantly higher than those in healthy control group, the above indexes were significantly lower in early and middle stage patients than those in advanced stage patients, and those in grade III and IV patients were significantly higher than those in grade I and II patients, suggesting that early diabetic renal damage can cause substantial pathological changes in the renal parenchyma, leading to increased renal parenchymal stiffness, which is consistent with the report by Wang et al.^17^ Studies have shown that the Young’s modulus of patients with chronic kidney disease is significantly higher than that of healthy volunteers, which also supports the conclusion of this study.^18^

The possible reason is that tissue elasticity is closely related to its pathological structure. The main pathological changes in DN include nodular diffuse glomerulosclerosis and interstitial fibrosis, and these secondary pathological changes in the kidney can lead to increased parenchymal stiffness.^19^ The findings of this study confirmed that Young’s modulus (E values) of the renal cortex and medulla in patients with diabetic nephropathy was positively correlated with urine protein, serum creatinine, and serum uric acid concentrations. A number of previous studies have found that the SWV value of the renal cortex in patients with chronic kidney disease is positively correlated with serum creatinine,^20^,^21^ all of which support the conclusion of this study. The more significant increase in renal Young’s modulus indicates more severe renal impairment, suggesting that renal SWE can serve as an auxiliary evaluation approach for renal impairment in diabetic patients.

### Limitations:

There are still some shortcomings in this study. The number of subjects included in this study was limited, so the conclusions drawn may not be very convincing. In addition, we only analyzed and discussed the cases included in our hospital, which may not be representative enough. We look forward to a multi-center study in the future to reach more comprehensive conclusions.

## CONCLUSIONS

The analysis of hemodynamics and SWE may be beneficial for guiding the diagnosis of DN and aiding in clinical assessment.

### Authors’ Contributions:

**WD** and **XL:** Designed and did statistical analysis & editing of manuscript, is responsible for integrity of research. **HW and HF:** Conceived designed and helped in data collection. **KY:** Literature search, Did data collection and manuscript writing. All authors read and approved the final manuscript.
